# Assessment of adequacy and appropriateness of pain management practice among trauma patients at the Ethiopian Aabet Hospital: A prospective observational study

**DOI:** 10.1186/s12873-023-00869-9

**Published:** 2023-08-17

**Authors:** Wondwossen Alemu Ayano, Atalay Mulu Fentie, Melaku Tileku, Tilahun Jiru, Shemsu Umer Hussen

**Affiliations:** 1 Department of Pharmacy, Addis Ababa Burn, Emergency and Trauma Hospital, Addis Ababa, Ethiopia; 2 Department of Pharmacology and Clinical Pharmacy, College of Health Sciences, Addis Ababa University, Addis Ababa, Ethiopia; 3 Department of Emergency Medicine and Critical Care, College of Health Sciences, Addis Ababa University, Addis Ababa, Ethiopia

**Keywords:** Acute pain, Adequacy of pain, Time of analgesia, Pain management, Trauma patients

## Abstract

**Introduction:**

Pain is unpleasant sensory and emotional experiences associated with actual and/or potential tissue damage. It is the most common and prevalent reason for emergency departments (ED) visits with prevalence over 70% in the world.

**Aim of the Study:**

The study aimed to assess the adequacy and appropriateness of pain management at Aabet Hospital, Addis Ababa, Ethiopia.

**Methods:**

A hospital-based prospective cross-sectional study was conducted at Aabet hospital from December 1, 2020 to March 30, 2021. Adult trauma patients having pain (at least score 1 on Numeric Rating Scale) with Glasgow Coma Scale score > 13 were eligible to participate in the study. The pain intensity was evaluated at the time of admission (o minute) and then at 60, 120, 180, and 240 minutes. The time of the first analgesics was registered. The adequacy and the appropriateness of the pain management were calculated through pain management index (PMI).

**Results:**

Two hundred thirty-two (232) participants were included in this study of which 126 (54.3%) were admitted due to road traffic accident followed by fall 44(19%). Only 21 (9.1%) study participants received the first analgesic treatment within 30 minutes while 27(11.6%) participants had no treatment at all within 240 minutes. The mean pain intensity score at admission was 5.55 ± 2.32 and reduced to 4.09 ± 2.69. Nearly half 110 (47.4%) of the study participants were treated inadequately (PMI (-) score). There was a weak and negative correlation between PMI and time to analgesia (r = − .159, p = 0.0001). The type of analgesia used, the time to analgesia, and the degree of pain may predict 65% of the variance in PMI score (R2 = 0.65, P = .001).

**Conclusion:**

From the results of this study, it can be concluded that acute pain in trauma patients was under and inappropriately treated.

## Background

Pain is “an unpleasant sensory and emotional experience associated with, or resembling that associated with, actual and potential tissue damage” [1]. It is the most common and prevalent reason for emergency departments (ED) visits with very high prevalence all over the world. Painful conditions cause over 70% of all ED visits in the United States of America (USA) and Canada. They were trauma pain followed by urologic pain, abdominal pain, and non-trauma musculoskeletal pain [2, 3]. Pain prevalence in ED in sub-Saharan Africa has been as high as 83% [4, 5].

Acute trauma pain has been the most neglected but yet the most deleterious to the health and well-being of injured patients [6]. It’s to curb this negligence towards pain in an emergency setting that the guidelines and researchers recommend pain as a vital sign in emergency care [3].

Guidelines and experts recommend the management of acute pain in less than 30 min (time to analgesia) but the mean time for most setting in ER was 78 minutes in the US and greater in low-income countries [5, 7–10]. Appropriate acute pain management depends on the accuracy of interpreting patients self-reported measures of pain with one-dimensional pain intensity evaluation scales like the Numerical Rating Scale (NRS) and the clinical decisions about which drug to administer to the patients depends on the assessment of the acute pain [11, 12]. The adequacy and appropriateness of pain management could be assessed through validated tools. Pain Management Index (PMI) is a tool that is used to compare a specific patient’s pain intensity to the adequacy of the prescribed analgesics according to the WHO (World Health Organization) pain management ladder [13, 14].

The right pain management requires knowledge about pharmacological properties of non-opioids and opioids analgesics as well as, the assessment of risks related to opioid diversion, abuse, and misuse [2, 7]. A systematic review and meta-analysis showed that pharmacist-led intervention in pain management contributed substantially to pain management, ensuring the rational use of medicine and resulting in reduced pain intensity [15]. Clinical pharmacists perform pain assessments, assess for substance use disorders, and develop individualized treatment plans in pain management which increase the quality of care [16, 17].

This study was therefore aimed at assessing the pain intensity of trauma patients, time to analgesia of the trauma patients, adequacy and appropriateness of the pain management. It would be an important milestone in informing all concerned bodies to device interventions and conducting further research regarding the acute pain management practice in an emergency setting in Ethiopia.

## Methods

This study was conducted at the triage of emergency medicine and critical care department of Aabet hospital in Addis Ababa, Ethiopia. AaBET Hospital is a part of St. Paul’s Hospital Millennium Medical College. It is an emergency dedicated center with level 3 trauma care. It provides emergency and critical care services, orthopedic, neuro-surgery, general surgery, and plastics surgery services. The emergency department has 60 beds and the overall hospital bed is 300. The hospital serves about 400,000 populations in Addis Ababa and surrounding. The rate of admission at the hospital’s ED is 41–55 patients per day (about 15,000 to 20,000 patients per year) [18].

The primary objective of this study was to assess the adequacy and appropriateness of pain management at Aabet Hospital, Addis Ababa, Ethiopia and the secondary objectives of this study were to assess time to analgesia for trauma patients, to assess pain intensity before and after analgesia administration to trauma patients and to assess the adequacy of acute pain management in accordance with the WHO pain management ladder among trauma patients admitted to Aabet hospital.

A hospital-based prospective cross-sectional study design was employed. The data were collected for a period of four months (from December 1, 2019 to March 30, 2021) and data from patients’ medical chart and pain outcome questioners were used for the study.

All adult patients admitted to triage of emergency Aabet Hospital during the study period fulfilling eligibility criteria were included. The inclusion Criteria were patients presented with injuries due to trauma, patients ≥ 18 years of age, stable patients (stabilized Airway, Breathing and Circulation), Glasgow Coma Scale score > 13 (on a 3–15-scale where 3 indicates no sign of neurological function and 15 is full neurological function), patients having pain (at least score 1) according to verbal rating scale, and patients willing to participate in the study. The exclusion criteria were cognitive and mental disabilities (identified in patients’ clinical records), patients who require cardiopulmonary resuscitation, endotracheal intubation, or transferring to intensive care units during data collection and patients who were addicted, alcoholic and had opiate abuse. The dependent Variables were adequacy and appropriateness of pain management.

The sample size required was calculated by using single proportion sample size formula (n = z^2^ pq/ E^2^) and the assumption is that the prevalence of the patients reporting pain in the ED is 80.1% [19], as well as 95% confidence interval, and 5% error. The Patients were entered sequentially in the study

n=$$\frac{(\text{Z}\text{a}/2)2\text{P}(1-\text{P})}{\text{d}2}$$= 245

(Where, n = minimum sample size required for the study, Z = standard normal distribution (Z = 1.96) with a confidence interval of 95% and a = 0.05, P = prevalence of pain in emergency department in Ethiopia (80.1%) [19] and d = level of precision or tolerable margin of error = 5%)

In the past three months there were N = 1542 trauma patients admitted to the triage of the Aabet Hospital which was < 10,000 for which correction formula was used and the sample size calculated to be (n_f_ =$\frac{\text{n}\text{*}\text{N}}{\text{n}+\text{N}}$≈211). Considering 10% contingency, the total sample size would be 232. Convenience sampling technique was used.

A carefully designed data collection tool that was derived from American Pain Society Patient Outcome Questionnaire (APS-POQ-R) for Quality Improvement of Pain Management in hospitalized patients was used to meet the designed objective [13, 20]. Numeric rating scale was used to assess the pain intensity. Each patient was asked severity of their pain on a scale from 0 to 10, where 0 is no pain and 10 the worst pain. Pain intensity evaluated at triage was indicated as “t0”. The pain intensity was evaluated prospectively at the time of admission in the ED (t0) and then at 60, 120, 180, and 240 minutes by the selected nurses.

The time sections were derived from Emergency Severity Index (ESI) of the triage category where severe cases were admitted to Red area (should be treated immediately), Orange area (treat the patient in less than 10 minutes), moderate to Yellow (treat the patients in less than 60 minutes) and mild cases to Green area (treat the patient within 240 minutes). The data collectors rated and recorded the pain intensity of the trauma patient. The time of the administration of the first analgesic was recorded; if the second dose of analgesia was administered, it should also be recorded. Any side effects correlated to the analgesic drugs delivered were recorded.

The PMI has been proposed as an auditable measure of the appropriateness for analgesic therapy. The PMI is a tool that tries to correlate an individual patient’s pain intensity to the appropriateness of the prescribed analgesics according to the WHO pain management ladder. The PMI is calculated by first giving scores to both the patient’s pain intensity and the class of the analgesic prescribed. Based on previous studies, the cutoff points used for pain intensity were 0 for no pain, 1 to 4 for mild pain, 5 to 6 for moderate pain, and 7 to 10 for severe pain. Accordingly, the absence of pain was scored as 0, mild pain as 1, moderate pain as 2 and severe pain as 3. In a similar manner, different potency of analgesic drugs prescribed were categorized as 0 if no analgesic drug was prescribed, 1 if a non-opioid analgesic was prescribed (for example, NSAIDs), 2 if a weak opioid analgesic was prescribed (for example, tramadol), and 3 if a strong opioid was prescribed (for example, morphine).

The PMI was calculated by subtracting the pain intensity or score from the analgesic level and ranged from − 3 (patient had severe pain but no analgesic used) to + 3 (patient experienced no pain but was taking morphine). Negative scores on the PMI were considered as indicators of inadequate pain management, and scores of 0 and greater was labeled as conservative indicators of adequate pain management.

Variables and database were coded, set and entered, cleaned, and analyzed using Statistical Package for Social Science (SPSS) version 26.0. Descriptive statistics included mean and standard deviation for continuous variables and frequency and percentage for categorical data were used to summarize socio-demographic and relevant characteristics of the study participants. The normality of data on pain intensity was measured by Kolmogorov-Smirnov tests. The pain intensity was analyzed in different sub-groups using one-way analysis of variance (ANOVA) repeated measures test. The pain intensity in different time sections will be analyzed by the repeated measure.

Pearson correlations were conducted to check relationship between patients’ characteristics and adequacy of pain. Multiples linear regression Analysis of PMI as dependent variable and type of analgesia administered, time to analgesia and pain intensity at admission as independent variables were used to assess the adequacy of pain management (p < 0.05).

The reliability of pain assessment scale was tested by Cronbach’s Alpha coefficient and found to be reliable with a value of 0.96. Prior to the actual data collection process, Pretest was done on 20 (in about 10% of the participants) trauma patients two weeks before the day of actual data collection and based on the results obtained from pre-test, amendment was made on the assessment tools and way of assessment based on the inputs found on pre-test. Eight data collectors (two pharmacists (B.Pharm), five BSc Nurses and one Clinical nurse), a supervisor (Emergency medicine critical care resident) and four card room workers were hired and the principal investigator provided two day training to the data collectors and supervisor to familiarize them on data collection instruments and on how to collect the necessary data from patient medical charts and how to conduct patient interviews. The supervisor was supervising data collectors and facilitates the daily activities. All filled checklists were reviewed for completeness and consistency on daily basis by the supervisor and principal investigator.

Prior to data collection, the full protocol of this study was submitted to Ethical Review Committee of AAU, School of Pharmacy, College of Health Sciences and received ethical clearance with reference number ERB/SOP/205/10/2020, and also permission for data collection was obtained from Aabet hospital. Each study participant was informed about the purpose of the study and its potential risk (time to be spent and those willing to participate were included. Confidentiality and privacy of study participants was ensured during the interview and all information accessed were kept and restricted from any access. Thus, identifiers like the name and address of the patient were not recorded in the data abstraction formats. Written and/or verbal informed consent was obtained from study participants for the interview and to extract data from their medical charts.

## Operational definition

### Acute pain

Pain that is of short duration (less than three months) and is reversible. It’s of sudden onset, occurs immediately after an injury which is usually severe in nature.

### Adequacy of pain management

scores of zero or greater on PMI or a decrease in pain score to < 4 and a decrease from triage pain score of ≥ 2 [13, 21].

### Adult

is a person older than or equal to 18 years of age.

### Chronic pain

Pain that is persistent and has been experienced for more than three months.

### Oligoanalgesia

describe the lack of adequate treatment of pain in terms of dosages and rapidity of administration of analgesics for ED patients.

### Opiophobia

the fear of the use of opioids by health care professional when the patient is eligible for the administration of opioids.

### Pain intensity

the severity of pain in traumata patients, which would be assessed by numerical rating scale.

### Pain management index (PMI)

a tool that tries to correlate an individual patient’s pain intensity to the appropriateness of the prescribed analgesics according to the WHO pain management ladder.

### Persistent pain

any pain that goes on for longer than would be expected after an injury or illness

### Survival event

the administration of the first analgesics within the observed 240 minutes

### Time to analgesia

time between admissions (0 hrs) of the patient to the hospital (from triage) and the first dose of analgesia.

### Trauma

physical injury due to road traffic accident, burn, falls, gunshot wounds, fight, collision or any other emergency that results in physical injury.

### Trauma center

a specialized hospital facility that is designed to provide diagnostic and therapeutic services for patients with major trauma injuries. It’s an emergency department with specialized services.

## Results

### Sociodemographic characteristics of the study participants

From 1252 study participants encountered during the study period, a total of 232 study participants were included for analysis. Nearly three-fourth (69.8%) of the participants were males. The mean age of the study participants was 35.53 years and more than half (55.6%) of them were in the middle adulthood age (36–59 years). Majority (31.9%) of the participants were Oromo. Nearly half (45.26%) of the study participants had a primary level of education and nearly half (46.1%) of the study participants were civil servants (Table 1**)**.

**Table 1:** Socio-demographic characteristics of trauma patients admitted at Aabet Hospital, Addis Ababa, Ethiopia from December 2020 to March 2021 (n = 232**)**

Variables		Frequency (N)	Percent (%)
Sex	Female	70	30.2
	Male	162	69.8
Age	18–35	76	32.7
	36–59	129	55.6
	≥ 60	27	11.6
Education status	Illiterate	34	14.6
	Primary	105	45.26
	Secondary	60	25.86
	Tertiary	33	14.22
Occupation	Civil servant	107	46.1
	Military	4	1.7
	Retired	16	6.9
	Self employed	80	34.4
	unemployed	25	10.7
Ethnicity	Oromo	74	31.9
	Amhara	56	24.1
	Tigrayan	18	7.8
	Other^1^	84	36.2

Other: Guraghe, Wolaita, Haddiya, Somale, Afar

### Baseline disease characteristics of the study participants

More than half of the study participants 126 (54.3%) were admitted in the trauma center due to road traffic accidents followed by falling down accident 44 (19.0%) and fighting 22 (9.5%) while other causes of trauma were reported in 34 (14.7%) of the study participants. The type of trauma mainly found during this study was a fracture in 39.2% of the study participants, followed by contusion and stretching in 27.2%, laceration and wounds in 22.4%, burn in 9.9% and other injures in 1.3% of the study participants. Upper and lower limbs were the most parts of the body where the feeling of pain was observed in 126 (54.3%) of the participants followed by the trunk and head, 72 (31%) and 31 (13.4%) of the participants, respectively (Table 2).

**Table 2:** Baseline disease characteristics of the trauma patients admitted at Aabet Hospital, Addis Ababa, Ethiopia from December 2020 to March 2021 (n = 232)

Variables		Frequency (N = 232)	%
The kind of trauma	Fracture	91	39.2
	Contusion and stretching	63	27.2
	Laceration and wounds	52	22.4
	Burn	23	9.9
	Other^1^	3	1.3
The cause of the trauma	Road traffic accident (RTA)	126	54.3
	Fall	44	19.0
	Fight	22	9.5
	Collision	6	2.6
	Other^2^	34	14.7
The site of pain	Upper and lower limbs	126	54.3
	Trunk	72	31.0
	Head	31	13.4
	Other^3^	3	1.3

_1_Deep cuts, pierces on the skin, concussions

_2_Fire, building collapse, operating machine

_3_ Neck, ear, eye

### Pain management practices

Among the 232 patients participated in this study only 21 (9.1%) patients had received the first analgesic treatment within 30 minutes while 27(11.6%) had no treatment at all within the study duration (240 minutes). Furthermore, only 3 (1.3%) patients were treated non-pharmacologically. Most of the study participants 72 (31%) received non-opioids 59 (25.6%) participants received weak opioids, and 37 (15.9%) participants received strong opioids. With regard to dual treatment approach, only 25 (10.8%) of participants received non-opioids and weak opioids followed by 6 (2.6%) participants who were treated with non-pharmacologic pain management method with non-opioids, and 3 (1.3%) patients received non-opioids and strong opioids in the four hours follow up. As summarized in Table 3, the most commonly prescribed analgesic was tramadol 87 (37.5%) followed by diclofenac 65 (31.9%) and paracetamol 41 (17.7%). The mean time of receiving the first analgesic was 94.7 minutes, with a range of 20–240 minutes.

**Table 3:** Specific types of analgesics drug given to the patients during the study period from December 2020 to March 2021 at Aabet hospital, Addis Ababa, Ethiopia (n = 232)

Type of specific drug given	Frequency (n)	%
No Drug	30	12.9
Paracetamol	10	4.3
Diclofenac	59	25.4
Tramadol	56	24.1
Morphine	17	7.3
Pethidine	20	8.6
Paracetamol and Diclofenac	6	2.6
Paracetamol and Tramadol	22	9.5
Paracetamol and Morphine	3	1.3
Diclofinac and Tramadol	9	3.9

**Fig. 1:**
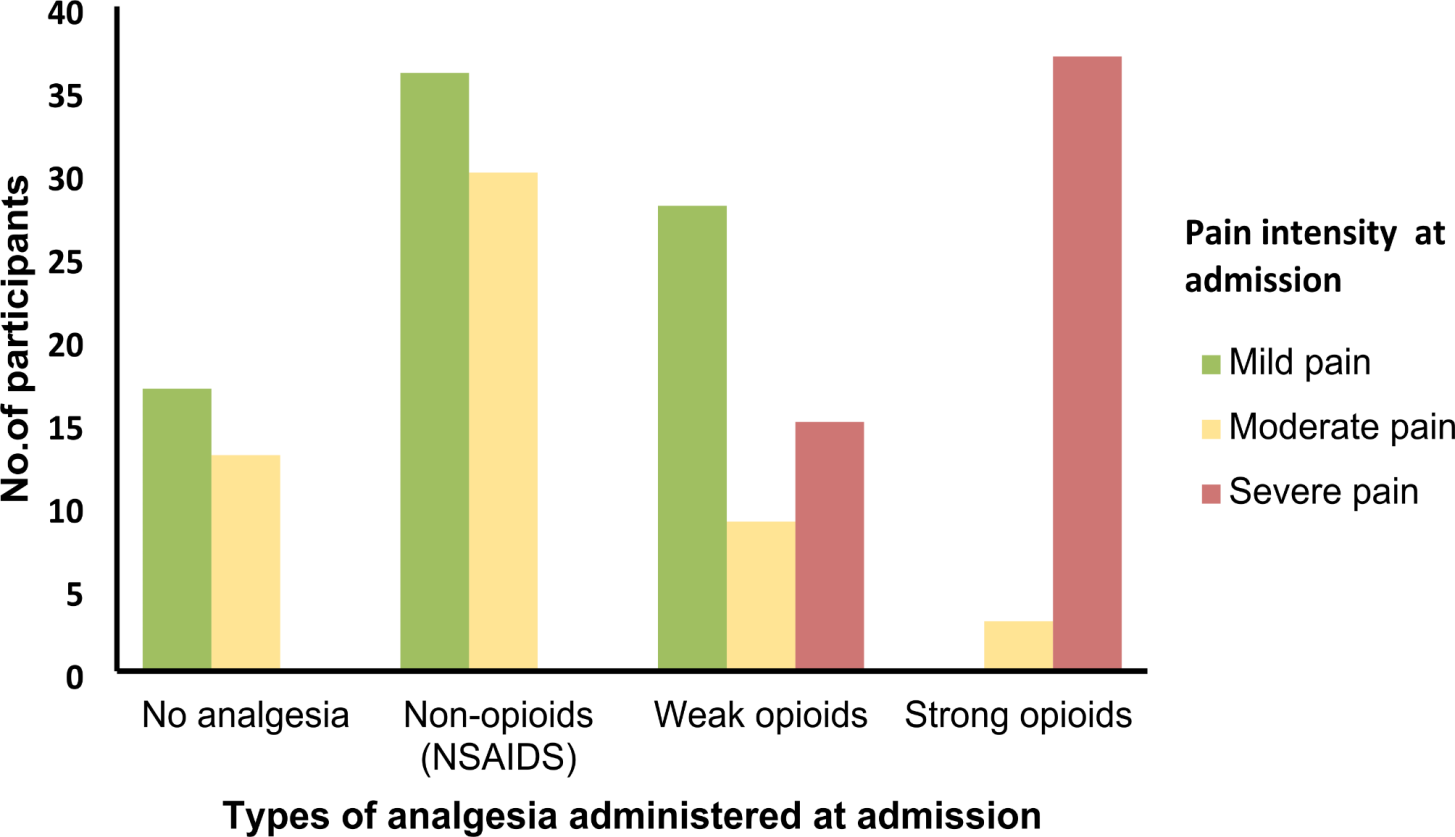
Types of analgesics administered according to pain score at admission at Aabet Hospital, Addis Ababa, Ethiopia, December 2020 (n = 232)

On the other hand, 17 (7.3%) participants with mild pain and 13 (5.6%) participants with moderate pain received no analgesia, 55 (23.7%) participants with severe pain received weak opioids and 3 (1.3%) participants with moderate pain received strong opioids as depicted (Fig. 1**)**

### Changes in pain intensity in trauma center

**Fig. 2:**
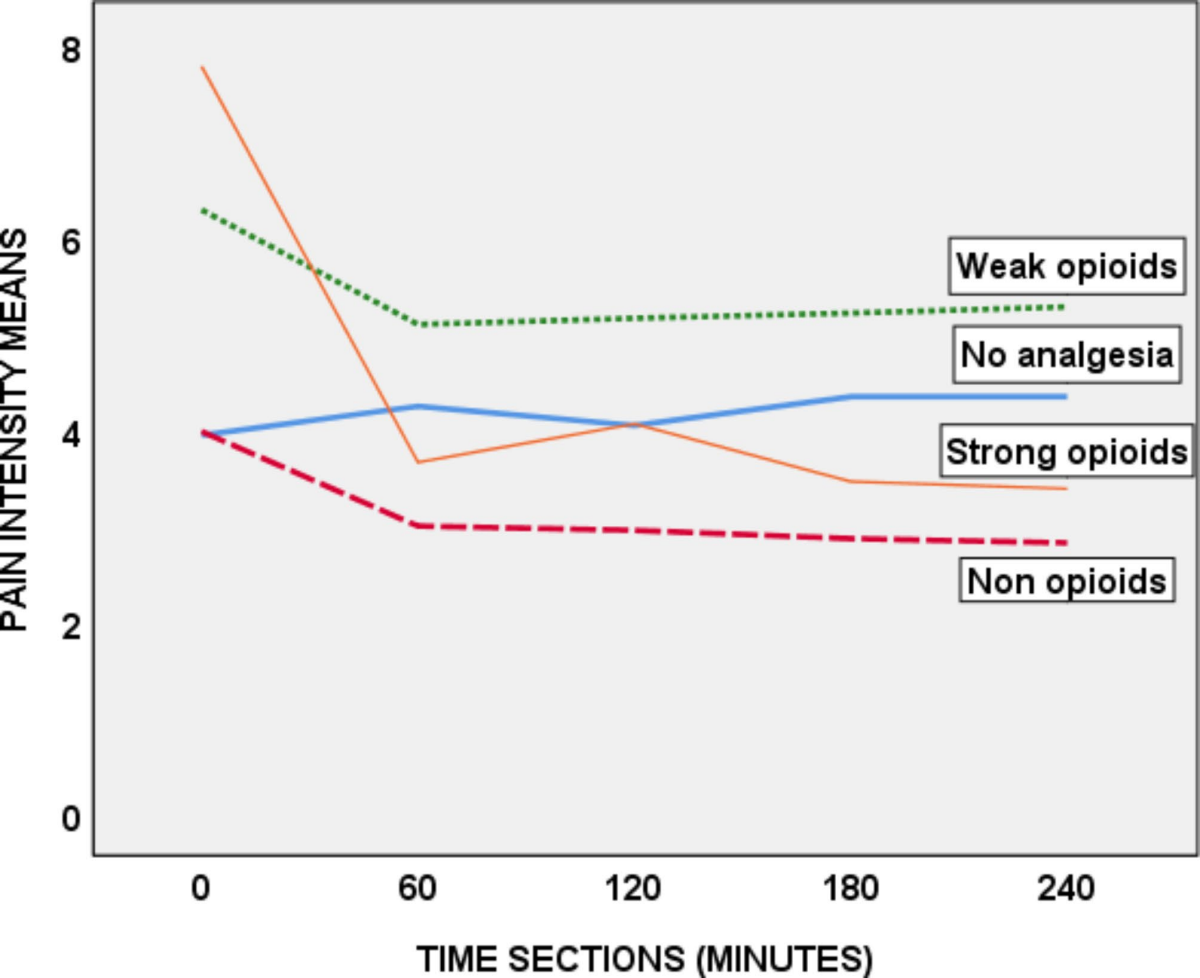
Mean pain Intensity trends of participants according to the analgesics given over 240 minutes Aabet hospital, Addis Ababa, Ethiopia from December 2020 to March 2021 (n = 232)

The mean pain intensity score at admission was 5.55 ± 2.32 while, it was reduced to 4.09 ± 2.69 at 240 minutes. Out of 232 patients encountered at admission, two-fifth 95 (40.9%) participants were in severe pain while nearly a quarter 55 (23.7%) of the study participants were in moderate pain, and 81 (34.9%) participants were with mild pain and. At the end of follow up (at 240 minutes), only 21 (9.1%) participants were in pain free while 103 (44.4%) participants were in mild pain, 44 (19%) and 64 (27.6%) were in moderate pain and in severe pain, respectively as shown in Table 4 and Fig. 2.

**Table 4:** Trends of Pain intensity of the trauma patients over 240 minutes at Aabet hospital, Addis Ababa, Ethiopia from December 2020 to March 2021 (n = 232)

Time pain intensity measured							
**Pain intensity**		0 min	60 min		120 min	180 min	240 min
		N (%)	N (%)		N (%)	N (%)	N (%)
	No pain (0)	0	0		6 (2.6)	9 (3.9 )	21 (9.1)
	Mild pain (≤ 3)	81 (34.9)	103(44.4)		101 (43.5)	118(50.9)	103 (44.4)
	Moderate pain (4–6)	55 (23.7)		88 (37.9)	73 (31.5)	47 (2.2)	44 (19)
	Severe pain (7–10)	96 (41.4)		41 (17.7)	52 (22.4)	58 (25)	64 (27.6)
	mean ± SD	5.55 ± 2.32		4.10 ± 2.10	4.16 ± 2.3	4.09 ± 2.51	4.09 ± 2.69

One-way ANOVA repeated measure test compared means of pain intensity for each of analgesic type administered over 240 minutes. It showed that the mean pain intensity of those receiving no analgesia increased from 3.93 to 4.33 and those receiving non-opioids, weak opioids and strong opioids decreased from 3.97 to 2.81, 6.28 to 5.27 and 7.78 to 3.38, respectively as illustrated in the following diagram.

### Adequacy of pain management and predictors of pain reduction

Among the patients admitted in the ED of Aabet hospital, almost half (110 (47.4%)) of them were treated inadequately having a PMI (-) score, of which nearly two-third (37.9%) were in moderate or severe pain. Pearson correlations were conducted to check associations between patients’ characteristics and adequacy of pain management. Statistical significance was determined at p < 0.05. Table 5 showed the linear correlations of PMI with different variables as all variables had statistically significant correlations except sex and age. There was a weak and negative correlation between PMI and time to analgesia. (Pearson correlation r = − .159, p = 0.0001). Correlation analysis revealed that pearson correlation r was found to be -0.159 which was between − 0.01 and − 0.29, i.e. Weak relationship. The negative r value of Pearson correlation indicated that as a time to analgesia was increasing, the PMI was decreasing, i.e., negative relationship.

**Table 5:** Associations of Pain Management Index (PMI) of patients at Aabet Hospital, Addis Ababa, Ethiopia from December to May 2021 with respect to the independent variables (n = 232**)**

Variable	Adequate Pain Management N(%)	Inadequate Pain Management N(%)	Total	P-value^1^
**Age(years)**				0.225
18–35	53(22.8)	76(32.8)	129	
36–59	45(19.4)	31(13.4)	76	
> 59	14(6)	13(5.6)	27	
**Sex**				0.953
Male	78(33.6)	84(36.2)	162	
Female	34(14.7)	36(15.5)	70	
**Pain Intensity at admission**				0.001
Mild	23(9.9)	58(21.1)	81	
Moderate	30(12.9)	25(10.8)	55	
Severe	59(25.4)	37(15.9)	96	
**Type analgesia used**				0.001
No treatment	27(24.1)	0	27	
Nonpharmacologic	3(2.7)	0	3	
Non opioids	20(17.9)	52(43.3)	72	
Weak opioids	37(33)	22(18.3)	59	
Strong opioids	0	37(30.8)	37	
**Time to analgesia**				0.015
0–30 min	3(2.7)	13(10.8)	16	
31–60 min	44(39.3)	51(42.5)	95	
61–90 min	31(27.7)	24(20)	55	
91–120 min	3(2.7)	4(3.6)	7	
121–180 min	4(3.6)	25(20.8)	29	
181–240 min	27(24.1)	3(2.5)	30	

^**1**^ Pearson correlation

In this study, multiple linear regression analysis (as depicted in Table 6) was employed and showed the type of analgesia administered, time to analgesia and pain intensity at admission had fairly strong relationship with PMI (R^2^ = 0.65, Adjusted R^2^ = 0.651, p = 0.0001) while the types of treatment (non-pharmacologic and pharmacologic) and the number of analgesia used were not significant at p < 0.05. The type of analgesia administered, time to analgesia and pain intensity could predict 65% of the variance in PMI score. As the time to analgesia increases by 1 minute, the PMI decreases by -0.001 when all other independent variables are held constant and as the pain at admission increases by one unit, the PMI decreases by -0.191 unit when all other independent variables are held constant and as one unit increase unit in the type of analgesic used or as the patient switched from weak opioids to strong opioids, the PMI increase by .514 units.

**Table 6:** Multiple linear regression Analysis of pain management index as dependent variable at Aabet hospital, Addis Ababa, Ethiopia, March 2021 (n = 232)

Variables	B^1^	Beta^2^	t	p-value	95% CI for B	
					Lower	Upper
Constant	.964		11.54	.0001	.616	1.034
Treatment given	− .024	-081.	− .966	.325	− .093	.034
Time to analgesia	− .001	− .101	-2.14	.034	− .053	.015
Pain at admission	− .191	− .884	-17.75	.0001	− .489	− .053
No. of analgesia	− .069	− .075	-1.10	.269	− .192	.095
Type of Analgesia	.514	.941	14.98	.0001	.397	.558

^1^ Unstandardized Coefficient

^2^ Standardized Coefficients

The type of analgesia administered, time to analgesia, pain intensity at 0 min(admission), and number of analgesia could predict 76.1% of the variance in the individuals’ pain score at the end of 240 minutes (R^2^ = 0.761,Adjusted R^2^ = 0.755,P = 0.0001) from multiple linear regression analysis as depicted in the Table 7. When all these independent variables were taken together and compared with the dependent variable (pain score at the end of 240 minutes) showed a strong correlation. (r = 0.873, P = .0001) between the dependent and independent variables were found. As the number of analgesics increase by one unit, the pain score decreases by -0.232 at the end of 240 minutes supporting multimodal pain management (p = 0.037). As the time to analgesia increases by one minute, the pain score at the end of 240 minutes decreases by -0.002 units. Other variables like age, sex, kind of trauma, cause of trauma and site of pain couldn’t predict pain reduction (p < 0.05).

**Table 7:** Multiple linear regression analysis of pain score at the end of 240 minutes as dependent at Aabet hospital, Addis Ababa, Ethiopia (n = 232)

Variables	B^1^	Beta^2^	t	p-value	95% CI for B	
					Lower	Upper
Constant	1.987		5.79	0.0001	1.309	2.665
Age of the patient	− .002	− .021	− .614	0.540	− .007	.004
Analgesic given	− .159	− .138	-2.42	0.016	− .289	− .030
Time to analgesia	− .002	.056	1.26	0.015	− .003	− .001
Pain at admission	.375	.821	16.30	0.0001	.330	.420
No.of analgesics	− .232	0.134	2.05	0.037	− .449	− .014

^1^ Unstandardized Coefficient

^2^ Standardized Coefficients

## Discussion

Despite being the most frequently reported compliant, acute pain has not been treated adequately and appropriately in ED [22]. Therefore, this study was carried out in the ED of Aabet hospital to assess acute trauma pain intensity, the time for the first analgesia after admission in the hospital, the adequacy and appropriateness of pain management. Most (126 (54.3%), of the participants admitted in the trauma center were due Road traffic accidents (RTA) followed by falling down accidents (44 (19%), fighting (22 (9.5%), and other kinds of trauma (34 (14.7%). This was in line with studies done in Australia, South Africa, Italy, and the United States of America [2, 23–26].

The findings of this work revealed that 11.6% of participants had no treatment at all within the 240 minutes. A study conducted in Nigeria showed that no preoperative analgesia was prescribed for 45.2% trauma and non-trauma patients and 77% of the patients considered the doses inadequate [10]. A multicenter study in US and Canada showed that about 40% of the study participants didn’t get analgesia after admission at ED [9]. Similarly, study conducted in Iran showed that 60.8% of the ED patients had not received pain analgesia during four-hour follow up [27]. Such variation could be due to the fact that present study was only among trauma patient at ED of trauma center where acute pain prevalence would be higher than other ED departments and increased the likelihood of receiving analgesia.

The present study showed that the most commonly prescribed analgesia was tramadol 87 (37.5%) followed by diclofenac 65 (31.9%), paracetamol 41 (17.7%) and morphine 17(7.3%). Consistent with the findings of this study, other research conducted in Gondar, Ethiopia revealed that 39.9% of patients received tramadol, 19.7% received diclofenac and less than 4.5% of the patients received any strong opioids [5] Similarly, study conducted in Yekatit 12 hospital, in Ethiopia showed that 87.1% of the patients were feeling severe pain but the patients were taking only non-opioids for their severe pain and none of them received strong opioids [28]. Likewise, a study conducted in western Kenyan hospital, 54.5% had been prescribed non-opioids, 17% had been prescribed weak opioids, and 14% had been prescribed strong opioids of all participants. But in contrast to our finding the most commonly prescribed analgesia was paracetamol 30.9% followed by tramadol 15.5% and diclofenac 14.5% [29]. In contrast a worldwide survey showed that preferred analgesia for management of acute pain were morphine, fentanyl and paracetamol with or without combination [30]. Accordingly, lower frequency of opioid analgesia was reported in this study and this discrepancy could be due to the fear of addiction (opiophobia), the costs and availability of strong opioids [4, 31–33].

Multimodal pain management approach has the ability to better control pain and reduces opioid consumptions and complements opioid, thereby opioid sparing [7, 34, 35]. Similarly, in this study, as the number of analgesics (combination of analgesia) administered to the participants increased, the pain score at the end of the 240 minutes decreased significantly (r= -0.871, p = .0001).

The present study showed that nearly half (110 (47.4%) of the participants were treated inadequately though 88 (37.9%) were in moderate or severe pain. Studies done in Gondar, Ethiopia had a comparable findings with the current study in that 57% of the patients reported that the analgesic was not adequate [5]. Study conducted in Nigeria showed that no preoperative analgesia was prescribed for 45.2% trauma and non-trauma patients and in patients who had preoperative analgesia, 40% of the patients considered the doses inadequate [10]. Consistent with this study prevalence of pain at discharge and inadequate pain control were found to be prevalent in studies done Australia, US and Canada [6, 23, 36, 37]. The finding of this work that revealed the prevalence of adequate analgesia to be 52.6% was consistent with the study in Australia which showed 58.7% of the participants got adequate analgesia [21]. Similarly, a prospective multicenter study in US and Canada by Todd et al indicated that pain and oligoanalgesia were very rampant and only 60% got analgesia [9]. In contrast, a prospective study in Iran on pain management of trauma patients in the emergency department showed that only 13.3% of the patients were given adequate analgesia [27].

Psychometric evaluation of the American Pain Society Patient Outcome Questionnaire showed a reduction of approximately 30% in a Numeric Rating Scale in acute pain has been considered as a clinically important difference [20]. Accordingly, the current study showed a reduction of pain intensity from 5.55 ± 2.32 (at admission) to 4.09 ± 2.69 at 240 minutes and revealed clinically important difference but yet inadequacy. Similar study in Iran showed the reduction of the average pain intensity score at admission was 6.16 ± 2.63 to 5.27 ± 2. Within four hours [27]

The inadequate pain management may emanate from several barriers in resource-limited setting. The limited availability and unaffordability of opioids are the major ones which pose a significant challenges to pain management [31]. Accesses to opioids have been restricted through bureaucratic laws despite the rational and appropriate need for opioids [33, 38]. Additionally, the knowledge and attitudes of the patient and the practitioners have impact on pain management [39].

It’s generally recommended that patients in ED should get their first analgesia within 30 minutes [1, 7, 9]. However, the prevalence of patients who received the first analgesia within 30 minutes in this study was only 9.1%. Study in Gondar, Ethiopia in trauma and non-trauma patients also revealed that only 12.3% of patients received analgesia within 30 minutes of ED presentation [5] A comparable finding (19.2%) was reported from a study done in Australia [40]. Nevertheless, a higher prevalence (61.3%) than the current study was obtained from another study done in Australia [41].

The present study found that mean time of the first analgesia was 94.7 minutes which was much higher than studies done in US, Canada, Australia, Iran and Netherlands in which the mean time of analgesia for most setting in ER was 78 minutes [7, 9, 27, 40–42]. Study in Gondar, Ethiopia in trauma and non-trauma patients revealed the mean time to delivery of analgesia was 61 minutes [5]. This disparity may be due to the fact that more than half of the participants (54.3%) in this study were admitted because of road traffic accident where the participants were requested to provide insurance information to get free analgesia and hence that process took longer time in addition to the registration process for admission. In addition there existed pain management protocol in the study setting but there was no implementation of acute pain protocol. The implementation of acute pain protocol shortened the time to analgesia of emergency patients [8, 43]. In contrast to this study setting which was emergency department of trauma center, in other hospital emergency setting proposed reason for lengthy delay of pain management in ED were the growing numbers of chronic diseases in the community and reduced access to primary healthcare and ED overcrowding which also holds true to current study setting [44].

Pain relief was not associated with time to analgesia but it had association with ED length of stay [21, 40, 41]. However, this study found that pain score at the end of 240 minutes was significantly related with the time to analgesia (p = 0.034). Multiple linear regression showed that the time to analgesia, the type of analgesia used, pain intensity at 0 min (admission), and numbers of analgesia could predict 76.1% of the variance in the individuals’ pain score at the end of 240 minutes (R2 = 0.761, Adjusted R2 = 0.755,P = 0.0001). When the other variables held constant one minute increase in time of analgesia would decrease the pain assessment score by 0.02%. The discrepancy could be due to when the analgesics were given near the pain assessment minutes, the lesser the reports of pain since in this study analgesia was given lately near 240 minutes pain assessment. Moreover, the value under standardized beta coefficient showed time to analgesia had the weakest and almost insignificant contribution to pain at the end of 240 minutes (Beta = 0.56, 0.31%).

This study found that time to analgesia had negative and weak correlation with PMI (r = − .159, p = 0.0001). Multiple linear regressions also revealed that time to analgesia, the type of analgesia used, and pain intensity at admission could predict 65% of PMI (R^2^ = 0.65, Adjusted R^2^ = 0.651, p = 0.0001). As the time to analgesia increased by 1 minute, the PMI decreased by -0.001 units when all other independent variables are held constant. That means when the analgesics were given near the pain assessment minutes, the lesser the reports of pain but the overall pain management adequacy was improved as early as the first analgesia as shown on the PMI. This study had also addressed the gap that strong opioids were not administered as early as NSAIDs and weak opioids despite the patients were in severe pain. This might be due to misconception and the fear of strong opioids for addiction (opiophobia), the lengthy bureaucracy to get strong opioids, costs and availability of strong opioids [31, 32, 45, 46].

The present study found that being in severe pain had the highest probability of getting early analgesia. A prospective study conducted in emergency department in northwest Ethiopia, Gondar had similar outcome with our finding where it revealed a patient presenting at ED with severe pain was 3.5 times more likely to receive analgesia compared to those with mild pain (AOR = 3.5, 95% CI 1.42–8.54) and trauma patients had higher probability of receiving early analgesia than non-trauma patients (AOR = 3.99, 95% CI 2.01–7.94) [5] Similarly, a retrospective study done in Australia revealed that time to analgesia were associated with moderate (OR = 2.73, 95% CI 2.13–3.49) and severe pain score (OR = 8.74, CI 5.63 to 13.57) [40].

This study found that acute pain in trauma patient admitted in ED were undertreated and ignored as the fifth component of vital signs. Several studies in Ethiopia, confirmed that pain in cancer, in preoperative and postoperative, and in burn patients had not been treated adequately or had not received pain intervention [5, 13, 19, 28, 47]

This paper shed light on the importance of the time to analgesia and appropriateness of the pain management in trauma patients. Indeed, this study may help in filling the gaps in ED’s pain management practices through stimulating appropriate analgesic agent prescribing patterns and promoting adherence to good clinical practice set by the WHO.

### Limitations of the study

This study has some limitations. This was a single centered study, based on a nonconsecutive convenience sample of patients. As such, the results of this study were found from trauma patients admitted at the ED of trauma center and may not be generalizable to other ED settings with different staffing profiles and case mixes.

Moreover, discharge pain medication information was unknown due to shortage of study period over a single patient and the patient were not completing the treatment in the hospital within the four-hour period which was helpful to know the length of hospital stay due to pain. Additionally, as the study was in trauma center, the trauma patient were most likely transferred to orthopedic surgical room, neurosurgery room, plastic and reconstructive surgery unit or intensive care unit after getting emergency medical service for longer stay and there may be loss to follow up in the process.

## Conclusion

From the findings of this study, it can be concluded that acute pain in trauma patients in the emergency department was undertreated and ignored as the fifth vital sign. Furthermore, this paper shed light on the significance of time to analgesia and the appropriateness of pain management in trauma patients.

## Data Availability

All the data is presented in the main manuscript. Raw data can be obtained through an email to the principal author.

## Abbreviations

AaBET: Addis Ababa Burn Emergency and Trauma Hospital
ED: Emergency Department
IASP: International Associations for the Study of Pain
NRS: Numeric Rating Scale
NSAIDS: Non-Steroidal Anti-Inflammatory Drugs
PMAS: Pain Medication Appropriateness Scale
PMI: Pain Management Index
RTA: Road Traffic Accident
SPHMMC: St Paul’s Hospital Millennium Medical College
SPSS: Statistical Package for Social Science
VRS: Verbal Rating Scale
WHO: World Health Organization
